# Reduction in abdominal hysterectomy surgical site infections: the impact of monitoring and feedback

**DOI:** 10.1017/ash.2025.10207

**Published:** 2025-11-27

**Authors:** Abegail Pangan, Sophie Labrecque, Michael Parry, Shweta Karki, Suzanne J. Rose, Asha K. Shah

**Affiliations:** 1 Stamford Hospital Department of Infection Prevention, One Hospital Plaza, Stamford, CT, USA; 2 Westchester Medical Center, Valhalla, NY, USA; 3 Stamford Hospital Department of Infectious Diseases, One Hospital Plaza, Stamford, CT, USA; 4 Stamford Health Department of Research and Discovery, One Hospital Plaza, Stamford, CT, USA

## Abstract

**Objective:**

This quality improvement project aims to contribute to the body of knowledge on effective and sustainable surgical site infection (SSI) prevention strategies in a teaching community hospital setting.

**Design:**

Retrospective and prospective chart review.

**Setting:**

305-bed acute care urban teaching hospital.

**Patients:**

All abdominal hysterectomies were included in the data analysis from January 1, 2018 to June 30, 2025.

**Methods:**

The project consisted of two time periods: baseline and monitoring periods. During the baseline period, an SSI prevention bundle was in place, but no individual bundle elements were monitored. At the end of the baseline period, due to rising SSI rates after abdominal hysterectomy, a multi-disciplinary workgroup was formed to evaluate bundle complexity and identify deficiencies in practice. Staff were re-educated and audit tools were created. In the monitoring period, compliance with individual bundle elements was measured and results were disseminated to stakeholders.

**Results:**

There were 782 abdominal hysterectomy procedures in the baseline period and 497 in the monitoring period. 13 patients had an SSI in the baseline period versus one patient in the monitoring phase (*p* = 0.003). Compared with the baseline, the SSI rate decreased significantly during the monitoring period (0.2 SSI versus 1.7 per 100 abdominal hysterectomies, *p* = 0.002).

**Conclusion:**

This project demonstrated significant improvements in outcome (SSI rates) when care processes were regularly monitored, and compliance was shared with key players. Future quality improvement projects will be focused on prioritizing the most important care processes that need to be continuously monitored.

## Introduction

Surgical site infections (SSIs) represent a significant and preventable form of healthcare-associated infection, posing substantial risks to patient safety and increasing healthcare costs.^1^ Among the various surgical procedures, abdominal hysterectomy is a major gynecological surgery with a notable risk of SSI, despite advancements in surgical techniques and prophylactic measures.^2^ The burden of SSIs extends beyond morbidity and mortality, leading to prolonged hospital stays, re-admissions, and substantial financial implications for both patients and healthcare systems.^3–5^

In recognition of their high prevalence and preventability, various coordinated and evidence-based strategies have been developed to mitigate SSI risk. The effectiveness of these guidelines has been widely documented in numerous studies, demonstrating a clear link between their implementation and a reduction in postoperative SSIs.^1,6,7^ The importance of these measures was further underscored by the Centers for Medicare and Medicaid Services (CMS), which incorporated SSI outcome measures for specific procedures, including abdominal hysterectomy and colon surgery, into its Hospital-Acquired Condition Reduction Program beginning in fiscal year 2016. This program mandates detailed data reporting and applies financial penalties to non-compliant institutions, creating a strong incentive for hospitals to reduce SSI rates and meet stringent quality targets.^8^

While short-term quality improvement initiatives have successfully demonstrated the effectiveness of these guidelines in reducing SSI rates,^9–14^ a significant challenge remains: the long-term sustainability of these gains. The literature, while robust in demonstrating the impact of guideline implementation, is less clear on the specific mechanisms and interventions required to sustain a culture of continuous improvement and maintain low SSI rates. This gap highlights a critical need for practical, scalable strategies that go beyond one-time interventions to ensure ongoing adherence to best practices using a multidisciplinary approach.

This quality improvement project sought to address this gap by focusing on the sustained reduction of SSIs following abdominal hysterectomies. The primary objective was to decrease the SSI rate by implementing a novel, multi-faceted, and multidisciplinary monitoring and feedback process. A secondary objective was to systematically document the key steps and interventions undertaken to ensure the long-term sustainability of the reduced SSI rate, thereby providing a replicable model for both our and other healthcare institutions. By describing the impact of a dedicated monitoring and feedback system, this quality improvement project aims to contribute to the body of knowledge on effective and sustainable SSI prevention strategies in a teaching community hospital setting.

## Study design and methods

Stamford Hospital is a 305-bed community hospital in southwestern Connecticut. Surgical and gynecology services are staffed by hospitalists and residents in combination, supervised by salaried hospital faculty and community-based surgeons and gynecologists. Surgical site infection rates for colorectal surgery and abdominal hysterectomies are reported monthly to the National Health and Safety Network (NHSN) as required by our participation in that program. NHSN definitions were used to determine SSIs.^15^ As part of a quality improvement effort, Institutional Review Board approval was not required and no individual patient identifiers were captured.

This project consisted of 2 periods: (1) a *baseline period* (January 2018–February 2022) where SSI rates following abdominal hysterectomies were measured without any monitoring of surgical processes; and (2) a *monitoring period* (March 2022–June 2025) where both process measures and SSI rates were monitored and results were reported regularly to stakeholders.

### Baseline period

**Figure 1.**
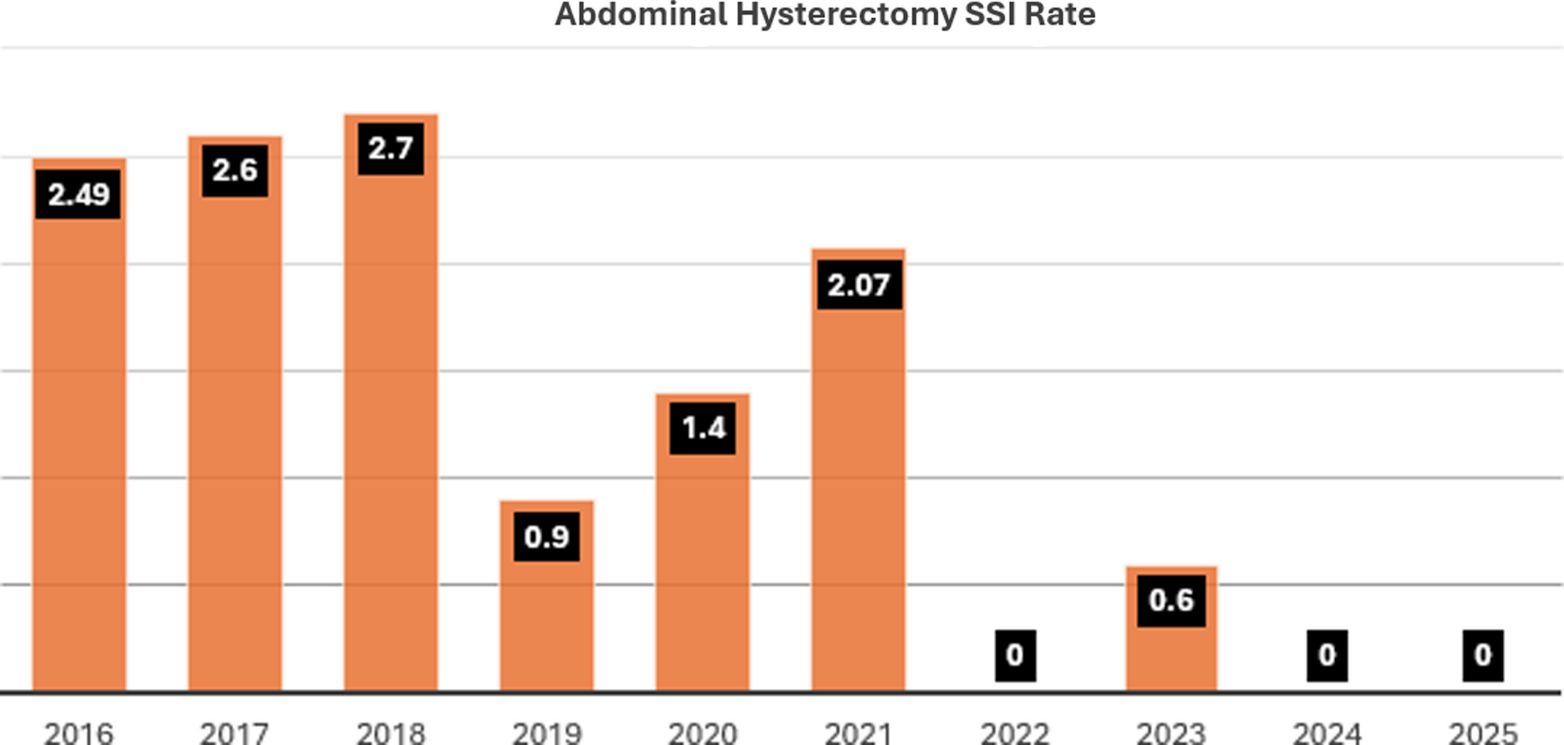
Distribution of SSI rate from 2016 to 2025. The prebaseline period extended from January 2016 to December 2017. The baseline period extended from January 2018 to February 2022 followed by the monitoring period from March 2022 to June 2025. All consecutive abdominal hysterectomies were included for analysis.

An increase in SSIs following abdominal hysterectomies was noted from 2016–2018 in the prebaseline period (Figure 1). An interdisciplinary workgroup was created, including representatives from Infection Prevention, Infectious Diseases, Perioperative Services, Obstetrics and Gynecology and Nursing. An SSI prevention bundle was implemented to standardize best practices and improve patient outcomes. The interventions implemented included a *preoperative* component that included chlorhexidine bathing and incisional skin preparation where the patient was instructed to shower the night before surgery with generic antibacterial soap. If not performed, the nurse would use 2% chlorhexidine wipes on the patients in the preoperative area. In addition, preoperative antimicrobial prophylaxis guidelines were rewritten to be consistent with national standards for abdominal hysterectomy.^16^

The *intraoperative* component of the bundle included appropriate technique and product for skin and vaginal preparation; decreasing wound contamination by using separate sterile closure tray; changing gloves after fascia closure; and closure with sutures rather than staples. The *postoperative* component included maintaining normal body temperature and providing postoperative education about patient hand hygiene and incision care.

Another essential element of the bundle was to provide education to patients about preoperative and postoperative measures to prevent SSI. A comprehensive brochure was developed for patient education and was given to every patient in the physician’s office prior to surgery. Once the SSI prevention bundle was implemented, the SSI rate was monitored but compliance with the interventions in the bundle (process measures) was not monitored. The implementation was successful as evidenced by the substantial reduction of SSI in 2019 (Figure 1).

However, between 2019 and 2021, the SSI rate subsequently increased (Figure 1) necessitating the multidisciplinary work group to evaluate the trend increase. Following a literature review, chart review and direct case observations, it was felt that interventions put in place in 2018 were still appropriate. However, it was suggested that gaps and inconsistencies existed in adhering to certain components of the practice bundle. The work group decided to focus on measuring adherence to the following elements of the current bundle: *intraoperative skin antisepsis and vaginal preparation, preoperative bathing, and antimicrobial prophylaxis.*

A re-education program was devised and a plan to measure bundle practices was created. The program included: (1) A video demonstrating proper technique for intraoperative skin antisepsis and vaginal preparation was presented to all Medical and Nursing staff; (2) Infection Prevention presented the current SSI prevention bundle at Obstetrics and Gynecology Grand Rounds and new resident orientation; and (3) SSI prevention bundle re-education for all relevant nursing staff.

**Figure 2.**
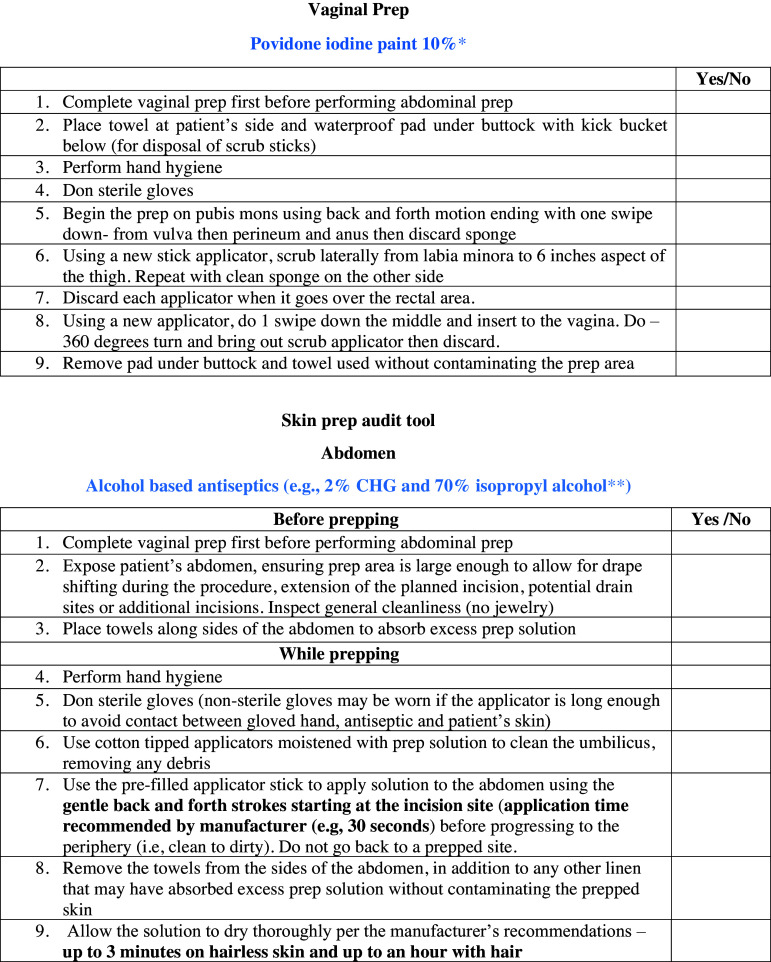
Abdominal and vaginal skin prep audit tool. Prep = preparation. **BD Chloraprep^TM^ and *Covidien Kendall Povidone iodine sponge sticks were utilized at our hospital.

Process measures began by implementing an audit tool (Figure 2) which was designed to directly observe skin and vaginal preparation practices in the operating room; immediate feedback was provided along with compliance rate. Subsequently, data was collected to evaluate staff compliance with documenting if the patient had showered at home preoperatively and if not, if patients were offered chlorhexidine bathing in the preoperative area. Finally, the medical record was audited to monitor antimicrobial prophylaxis.

### Monitoring period

Starting in March 2022, Infection Preventionists and members of Perioperative team started observing skin preparation and vaginal preparation antisepsis in the operating room. Audits and chart review were done retrospectively for antibiotic prophylaxis, preoperative home shower, and preoperative chlorhexidine wipes. There were 30 chart reviews per quarter randomly selected by convenience and 15 actual observations per quarter randomly selected by convenience (each case has 18 steps to be observed). Real-time feedback was given to staff. Medical record reviews were performed to monitor compliance with antibiotic prophylaxis and preoperative chlorhexidine bathing. Compliance rates were provided concurrently to all stakeholders quarterly, including departmental leadership. A goal of 90% and above was set for all process measures.

### Statistical analysis

Patient specific data were reviewed retrospectively and prospectively in the electronic medical record as a quality improvement project and as a function of patient record review during routine infection prevention activities. Risks factors were analyzed in the two groups to determine if demographic differences could explain the varying SSI rates. The risks evaluated were age, body mass index (BMI), duration of surgery, use of laparoscope, diabetes mellitus, and postoperative oncology diagnosis.

All consecutive abdominal hysterectomies were included in the analysis to assess the SSI rates and risk factors from January 1, 2018 to June 30, 2025. Surgical site infection rate was calculated by dividing the number of SSIs by the total number of procedures and multiplying by 100 (Figure 1). Surgical site infection rate and risk factors were compared between the baseline period and the monitoring period by univariate analysis using two-sample t test with unequal variance for continuous variables and Pearson’s χ^2^ test for categoric variables. Process measures were compared between the baseline period and the monitoring period by univariate analysis during Pearson’s χ^2^ test for randomly selected by convenience charts, as described above. The Pearson correlation test was used to assess the correlation with SSI rate and compliance rate. P-values < 0.05 were considered significant

## Results

1,279 abdominal hysterectomies were performed between January 1, 2018 and June 30, 2025. There were 782 abdominal hysterectomy procedures in the baseline period (January 2018–February 2022) and 497 in the monitoring period (March 2022—June 2025). The mean age of patients in baseline period was 53 years compared to 55 years in monitoring period (*P* = 0.04).

Risk analysis showed that there were more patients with diabetes mellitus during the monitored period compared to the baseline period (10.2% vs 14.4%, *P* = 0.04). The average ASA (American Society of Anesthesiologists Physical Status Classification System) in baseline phase was 2.2 compared to 2.3 in monitoring phase (*P* = 0.02). The percentage of laparoscopic surgery was significantly higher in monitoring phase than baseline (15.7% vs 10.2%) (*P* = 0.04). No other risk differences were detected (Table 1). 13 patients had a SSI in the baseline period compared to one patient in the monitoring phase (*P* = 0.003). Of the 13, 8 were classified as Organ/Space, 4 deep and 1 superficial SSI were detected on readmission to facility. Compared with the baseline, the SSI rate decreased significantly during the monitoring period (0.2 SSI vs 1.7 per 100 abdominal hysterectomies, *P* = 0.002).

**Table 1.** Patient characteristics comparison between baseline and monitoring phase (n = 1,279)

Characteristics	Baseline phase	Monitoring phase	
	Jan 2018–Feb 2022	Mar 2022–Jun 2025	
	(*n* = 782)	(*n* = 497)	*p*-value
Average SSI rate (no. SSI/procedures) * 100	1.7	0.2	0.002
**No. of SSI**	13	1	0.003
**Age, years (mean, sd)**	53.5 (12.4)	55.0 (12.9)	0.04
**Body Mass Index (kg/m** ^2^ **) (mean, sd)**	29.5 (6.8)	29.3 (7.2)	0.53
**ASA classification (mean, sd)**	2.2 (0.5)	2.3 (0.5)	0.03
**Duration of surgery, minutes (mean, sd)**	150.9 (64.5)	150.2 (53.4)	0.83
**Laparoscopic surgery**	82 (10.5%)	78 (15.7%)	0.006
**Diabetes diagnosis**	80 (10.2%)	70 (14.1%)	0.04
**Postoperative oncology diagnosis**	161 (20.6%)	83 (16.7%)	0.08

SSI, surgical site infection; kg, kilograms; m^2^, meters squared; ASA, American Society of Anesthesiologists Physical Status Classification System; sd, standard deviation. All consecutive abdominal hysterectomies were included for analysis.

**Figure 3.**
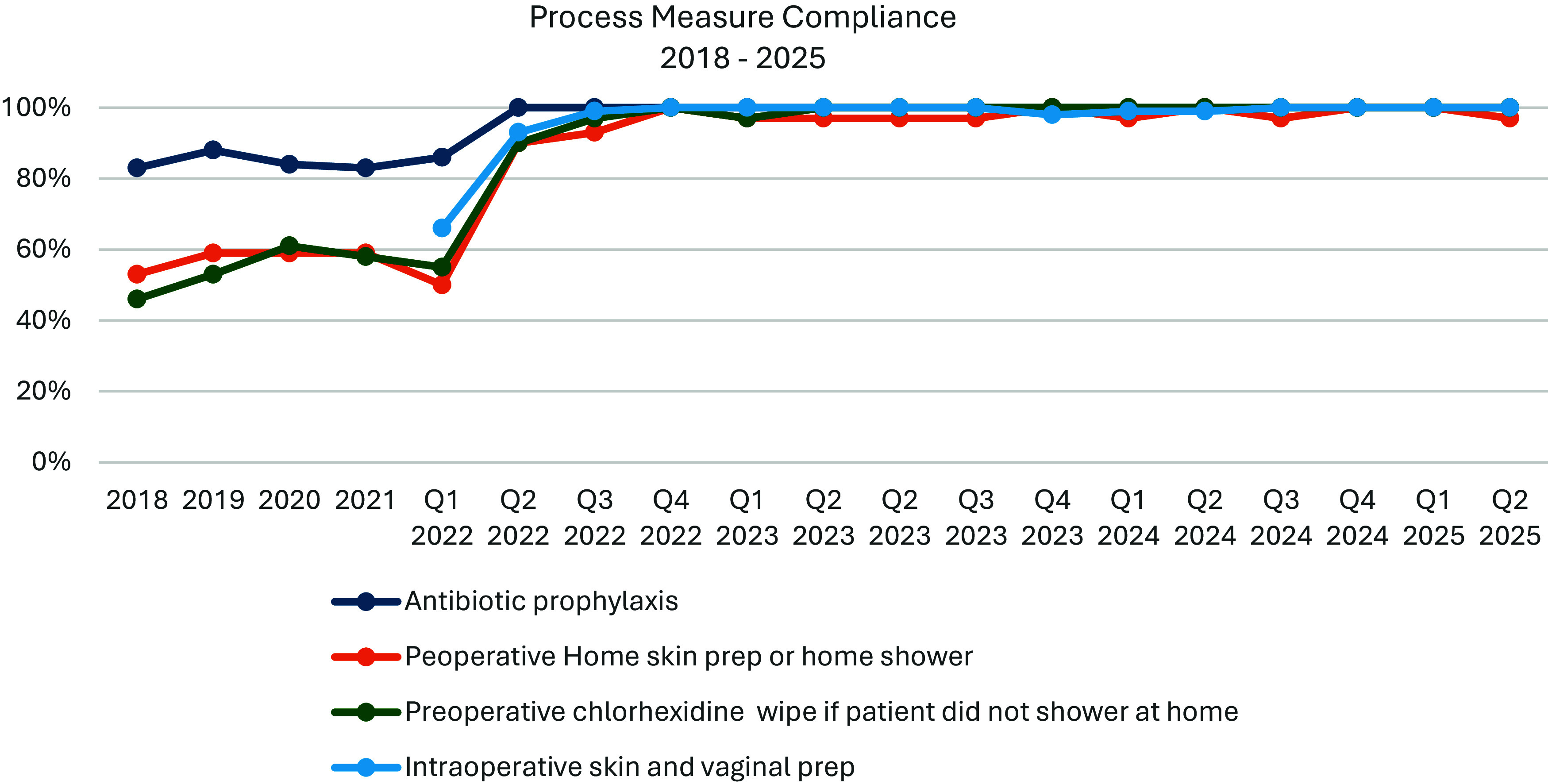
Process measures compliance rate (Baseline Jan 2018-Feb 2022 and monitoring period Mar 22-June 25). The baseline period collected yearly compliance data while the monitoring period collected and reported on quarterly compliance data. There were 30 randomly selected by convenience chart reviews per quarter for antibiotic, preop skin prep, preop CHG and 15 actual randomly selected by convenience observations per quarter done (each case observation has 18 steps to be observed).

At baseline, compliance rates for preoperative bathing or chlorhexidine wipes were below 60% and compliance with antibiotic prophylaxis standards was only 85% (Table 2). All the process measures in the monitoring period, including surgical skin and vaginal prep performance exceeded 90%. These rates have been sustained with continued monitoring and feedback for the study duration (Figure 3). When assessing the correlation between SSI rate and compliance for antibiotic prophylaxis and preoperative bathing or chlorhexidine wipes, the Pearson correlation coefficient showed moderate negative linear correlation (*r* = −0.6 and *r* = −0.5 respectively) a finding that was statistically significant (*P* < 0.05) (Table 3).

**Table 2.** Process measures comparison between baseline and monitoring phase

Process measures	Baseline Phase	Monitoring Phase	
	Jan 2018 -Feb 2022	Mar 2022 -Jun 2025	
	% compliance	% compliance	*p*-value
**Antibiotic prophylaxis (# charts reviewed)**	84.6% (*n* = 479)^^^	100% (*n* = 390)^^^	<0.001
**Preoperative home skin prep or home shower (# charts reviewed)**	57.2% (*n* = 479)	95.0% (*n* = 390)	<0.001
**Preoperative chlorhexidine wipes if patient did not shower at home (# charts reviewed)**	54.1% (*n* = 479)	96.7% (*n* = 390)	<0.001
**Intraoperative skin and vaginal prep (# steps observed)**	66.0% (*n* = 72)	99% (*n* = 3 150)*	<0.001

^^^Audit and chart reviews were randomly picked by convenience. There were 30 randomly selected by convenience chart reviews per quarter for antibiotic, preop skin prep, preop CHG and 15 randomly selected by convenience actual observations per quarter done (each case observation has 18 steps to be observed).

* For intraoperative skin prep and vaginal observations, there are 18 questions per audit tool.

**Table 3.** Pearson correlation between compliance rate and SSI rate

Process measures	SSI rate	
	(Pearson correlation coefficient = *r*)	*p*-value
**Antibiotic prophylaxis (# charts reviewed)**	–0.6	0.005
**Preoperative home skin prep or home shower (# charts reviewed)**	–0.5	0.02
**Preoperative chlorhexidine wipes if patient did not shower at home (# charts reviewed)**	–0.6	0.01
**Intraoperative skin and vaginal prep (# steps observed)**	0.10	0.72

SSI, surgical site infection. There were 30 randomly selected by convenience chart reviews per quarter for antibiotic, preop skin prep, preop CHG and 15 actual randomly selected by convenience observations per quarter done (each case observation has 18 steps to be observed).

## Discussion

In this study, we demonstrate that SSIs after abdominal hysterectomy were significantly reduced during a period of continuous monitoring and feedback compared to the baseline period where interventions were implemented but not monitored. While the monitoring period of this project did not introduce any new interventions from the original interventions during the baseline period, it was highly successful, highlighting the critical role of a continuous, data-driven monitoring and feedback system in achieving long-term sustainability. In addition, the observed significant increase in compliance rates for key process measures directly correlates with the reduction in SSIs.

Our findings are consistent with others demonstrating that implementing evidence-based care bundles can significantly lower SSI rates.^9–14^ However, these evidence-based bundles can be difficult to sustain due to workforce turnover, communication challenges and variation in workflows.^17^ Our research provides a key contribution to the literature in addressing the challenges of sustaining these improvements. Maintaining continuous surveillance can be highly successful^18^ in addition to hardwiring steps into protocol or order sets to sustain improvements that may otherwise erode over time.^17^

A key strength of this project is that the SSI rate decreased despite a shift toward a higher-risk patient population in the monitoring period, characterized by more patients with diabetes mellitus and a higher mean ASA score. The significant increase in laparoscopic cases also did not lead to a rise in SSIs, which further reinforces that the observed improvement was a result of the intervention rather than a change in surgical approach or patient risk. This suggests that adherence to the bundle elements successfully mitigated these inherent risk factors, strengthening the argument that the intervention, not external patient factors, was the primary driver of the improved outcomes.

Despite these positive outcomes, this project has several limitations. As a single-center, quality improvement study, the results may not be generalizable to other hospital settings with different patient populations, surgical volumes, or institutional cultures. In addition, there may be other risk factors that could have contributed to the development of SSIs as we relied on the risk factors identified by NHSN.^15^ Of note, the incidence of diabetes mellitus was more common during our monitoring period, a fact that might have increased our SSI risk. Finally, it is possible that patients sought care for SSIs outside of our hospital system during the monitoring period and were not captured in this data analysis. However, the clear temporal association and the reversal of the SSI trend following the re-education and monitoring periods provide robust support for the intervention’s efficacy.

In conclusion, this project showed significant improvements in outcomes when the interventions were monitored regularly and consistently. When compliance is shared with the key-players beyond measuring the outcome, a valuable, replicable model for healthcare institutions seeking to move beyond short-term quality improvement gains and establish a culture of continuous oversight can be created. Further investigation will be focused on prioritizing the most important care processes that need to be continuously monitored.

## Research transparency and reproducibility

All data is publicly available at https://figshare.com/s/bfdf2fec95f702d7a489.
